# Association between serum C-reactive protein and low muscle mass among US adults: Results from NHANES 1999 to 2006

**DOI:** 10.1016/j.clinsp.2025.100588

**Published:** 2025-02-01

**Authors:** Ruzheng Lin, Ying Chen, Kai Liu

**Affiliations:** a Department of General Practice, Hainan General Hospital, Hainan Affiliated Hospital of Hainan Medical University, Haikou, China; b Medical Laboratory Center, Hainan General Hospital, Hainan Affiliated Hospital of Hainan Medical University, Haikou, China; c Geriatric Center, Hainan General Hospital, Hainan Affiliated Hospital of Hainan Medical University, Haikou, China

**Keywords:** Inverse J-shaped, C-reactive protein, Low muscle mass, Weighted logistic regression, NHANES

## Abstract

**Introduction:**

Inflammation is hypothesized to be a pivotal factor influencing muscle function, with C-Reactive Protein (CRP) serving as a common biomarker of inflammation. However, the literature pertaining to the relationship between CRP and muscle mass remains scant, particularly among representative adult populations in the United States. The present study aimed to delve into the association between serum CRP levels and muscle mass among American adults, leveraging data from the National Health and Nutrition Examination Survey (NHANES) conducted from 1999 to 2006.

**Methods:**

A cohort of 13,518 participants, representative of the US adult population, underwent dual-energy X-Ray Absorptiometry (DXA) to assess Appendicular Skeletal Muscle Mass (ASM) and had their CRP levels measured. Muscle mass was defined using ASM adjusted by Body Mass Index (ASM/BMI) criteria. Employing weighted logistic regression models, restricted cubic spline analysis, and subgroup analyses, the authors examined the association between serum CRP and low muscle mass.

**Results:**

After meticulously adjusting for various covariates, the present findings revealed a positive association between serum CRP levels and the risk of low muscle mass in American adults (OR = 1.07, 95 % CI 1.01‒1.14, *p* = 0.016). Notably, an inverse J-shaped relationship was observed, with serum CRP inflection points of 0.273 mg/dL for the overall population, 0.172 mg/dL for males, and 0.296 mg/dL for females. Subgroup analysis further demonstrated that factors such as gender, race, educational level, smoking status, congestive heart failure, stroke, renal weakness/failure, coronary heart disease, diabetes, hypertension, vigorous physical activity, moderate physical activity, and muscle strengthening activities did not significantly impact this positive correlation (all p for interaction values > 0.05).

**Conclusions:**

This nationally representative cross-sectional study provides robust evidence of an inverse J-shaped association between serum CRP levels and the risk of low muscle mass in adults in the United States, with a critical inflection point of 0.273 mg/dL. These findings may inform future research and clinical strategies aimed at mitigating the negative effects of inflammation on muscle mass and function in the adult population.

## Highlights

• NHANES data from 1999 to 2006 show a positive link between serum CRP levels and low muscle mass risk in US adults.
• An inverse J-shaped pattern emerges, indicating a non-linear association between CRP levels and low muscle mass risk, with critical points at 0.273 mg/dL for the overall population, 0.172 mg/dL for males, and 0.296 mg/dL for females.
• This nationally representative study underscores the crucial role of inflammation in muscle function and advocates for future research and clinical strategies to address inflammation's impact on muscle mass and function.

## Introduction

The decline in muscle mass and strength is a common disease state, especially among middle-aged and elderly people.^1^ With the gradual loss of muscle mass and function, a decline in physical activity occurs, gradually leading to a decrease in quality of life.^2^ The decline in muscle mass and strength has emerged as an urgent medical concern, given its associated increase in the risk of falls and injuries, often leading to prolonged medical care and substantial treatment costs.^3^ This situation imposes a significant burden on societal resources and families.^4^ Recently, there has been growing research interest in the role of inflammation in the pathogenesis of low muscle mass.^5^ Inflammation regulation is considered a key factor influencing the progression of muscle atrophy.^6^ Additionally, age-related changes and alterations in lipid metabolism are associated with increased systemic inflammation.^7^ These factors not only contribute to the development and progression of decreased muscle mass but also play a role in the pathogenesis of various age-related diseases, such as type 2 diabetes,^8^ osteoarthritis,^9^ and cardiovascular diseases.^10^

C-Reactive Protein (CRP) is a common inflammatory marker that plays a crucial role in assessing tissue damage and acute inflammatory responses.^11^ It not only reflects the acute inflammatory response of the body to environmental factors such as infection and trauma but also provides valuable diagnostic clues for chronic diseases like arthritis and cardiovascular diseases.^12^ In recent years, with deeper research, increasing evidence suggests a close association between CRP and muscle health.^13^ Studies indicate a significant negative correlation between CRP and hs-CRP (high-sensitivity C-Reactive Protein) levels and muscle strength, suggesting that the inflammatory response may be one of the key factors affecting muscle function.^14^ However, in the context of low muscle mass, a disease closely associated with aging, it is imperative not only to monitor changes in muscle strength but also to pay attention to the reduction in muscle mass and decline in function, involving more complex physiological processes. It is worth noting that although some studies have explored the relationship between CRP and muscle wasting syndrome,^15^ these studies are mainly focused on Asian populations, with limited research conducted on adult populations in the United States. Given the differences in genetics, diet, and lifestyle among different populations, it is not appropriate to simply extrapolate the results of Asian studies to adult populations in the United States. Furthermore, questions remain regarding the nature of the relationship between adult serum CRP and low muscle mass, as well as how CRP serves as a predictive indicator for low muscle mass in different populations. These issues require further investigation and discussion.

To address this gap, the authors utilized data from the National Health and Nutrition Examination Survey (NHANES) conducted between 1999 and 2006, which provides a nationally representative sample of adults in the United States, to explore in-depth the relationship between serum CRP level and the risk of low muscle mass. This study aims to enhance understanding of the pathogenesis of low muscle mass and provide important scientific evidence for future prevention and treatment strategies.

## Methods

### *Study population*

**Fig. 1:**
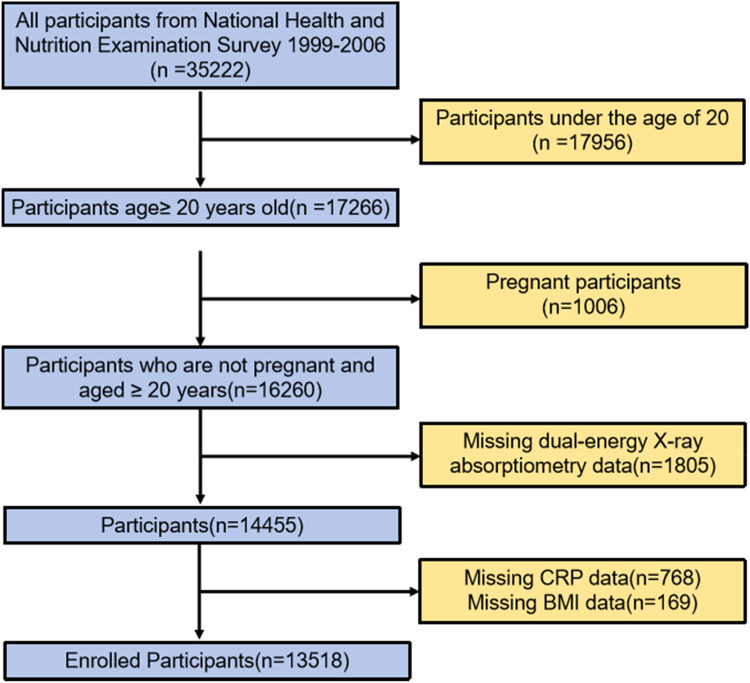
The flow chart for the present study.

NHANES in the United States is conducted by the National Center for Health Statistics (NCHS) of the Centers for Disease Control and Prevention (CDC).^16^ All data in NHANES are collected by trained professionals through household interviews or examinations in mobile examination centers.^17^ The survey includes cross-sectional interviews, examinations, and laboratory data collected from a complex, multi-stage, stratified, clustered probability sample representing the U.S. population. The survey protocol has been approved by the Institutional Review Board of the CDC. Informed consent was obtained from all participants. The protocol for this study was approved by the NCHS Ethics Review Committee (Protocol #98-12, Protocol #2005-06), and all participants provided written informed consent. Based on data from four consecutive NHANES cycles (1999‒2000, 2001‒2002, 2003‒2004, and 2005‒2006), the relationship between CRP and the risk of low muscle mass was established among US adults aged ≥ 20 years (Fig. 1). Among the 35,222 participants, 17,956 were excluded due to age < 20 years, 1006 were excluded due to pregnancy, 1805 had missing Dual-energy X-Ray Absorptiometry (DXA) data, 768 had missing CRP data, and 169 had missing BMI data. Finally, 13,518 participants were included in the analysis. This cross-sectional study follows the STROBE Statement.

### *Assessment of low muscle mass*

Measure the Appendiceal Skeletal Muscle Mass (ASM) using a dual-energy X-Ray Absorptiometry (DXA), which is the sum of the lean masses of the arms and legs. In this study, low muscle mass index was calculated as ASM adjusted by Body Mass Index (ASM/BMI), and males were classified as having low muscle mass if ASM/BMI < 0.827 and females < 0.518, based on the criteria designated by Guidance for the assessment of the muscle mass phenotypic criterion for the Global Leadership Initiative on Malnutrition (GLIM) diagnosis of malnutrition.^18^

### *Detection of serum C-reactive protein*

Blood samples are processed, stored, and transported to the University of Washington for analysis. The method used to determine C-Reactive Protein (CRP) is the latex-enhanced turbidimetric assay. Particle-enhanced assays are based on the reaction between a soluble analyte and the corresponding antigen or antibody bound to polystyrene particles. To quantitate CRP, particles composed of a polystyrene core and a hydrophilic shell are used to covalently attach anti-CRP antibodies. A diluted solution of the test sample is mixed with latex particles coated with mouse monoclonal anti-CRP antibodies. CRP present in the test sample will form an antigen-antibody complex with the latex particles. Automatic blank subtraction is performed. The CRP level is calculated using a calibration curve. Data reduction of the signals is performed using a storable logit-log function of the calibration curve. These assays are conducted on a Behring Nephelometer for quantitative CRP measurement (NHANES 1999‒2000: C-Reactive Protein (CRP) Data Documentation, Codebook, and Frequencies, cdc.gov).

### *Covariates*

After evaluating the relationship between CRP and low muscle mass in the elderly, selected and controlled risk factors related to low muscle mass. The following covariates were collected and considered: Age, Gender, Race, Education level, Marital Status, Smoking, Alcohol use, Hypertension, Diabetes, Asthma, Arthritis, Congestive heart failure, Coronary heart disease, Stroke, Emphysema, Thyroid disease, Chronic bronchitis, Liver disease, Cancer, Weak/failing kidneys, Vigorous activity, Moderate activity, Muscle strengthening activities, Family poverty income ratio, Body Mass Index(BMI), Systolic pressure, Diastolic pressure, Waist Circumference. The BMI was calculated by dividing the body weight by the square of the height. Diabetes diagnosed with an FPG equal to or greater than 7 mmoL/L or self-reported current use of anti-diabetic drugs. Hypertension was defined based on prior diagnosis, current use of antihypertensive medication, or systolic pressure/ diastolic pressure > 130/80 mmHg.^19^ The family poverty income ratio was defined as the family income to poverty line ratio after adjusting for inflation and family size. The selection of covariates was based on previously published studies and available variables.^18^^,^^20^^,^^21^

### *Statistical analysis*

Due to the complex design of the NHANES survey, the authors adhered to the NHANES recommendations and appropriately utilized four-year cycle weights, stratification, and clustering in the present study. Continuous variables were presented as the mean ± Standard Deviation (SD) (normal distribution), or the median (interquartile range) (skewed distribution). Since the sample size is greater than 5000, the authors used the Anderson-Darling test to assess the normality of the data. Categorical variables were presented as the number (percentage). Differences in baseline variables were tested using a weighted *t*-test, weighted Chi-Square test, or Fisher's exact test. For skewed distributed continuous variables, between-group comparisons are conducted using the weighted Wilcoxon rank-sum test.

A weighted logistic regression model was used to investigate the relationship between CRP and hypertension. Model 1 was not adjusted for any covariates. Model 2 was adjusted for age and gender. Model 3 was adjusted for age, gender, race, education level, marital status, smoking, alcohol use, hypertension, diabetes, asthma, arthritis, congestive heart failure, coronary heart disease, stroke, emphysema, thyroid disease, chronic bronchitis, liver disease, cancer, weak/failing kidneys, vigorous activity, moderate activity, muscle strengthening activities, family poverty income ratio, systolic pressure, diastolic pressure, waist circumference, white blood cell count, lymphocyte number, neutrophils, red cell count, hemoglobin, platelet count, red cell distribution width, albumin, alanine aminotransferase, aspartate aminotransferase, creatinine, triglycerides, glucose, serum cholesterol, blood urea nitrogen, total calcium, globulin, potassium, sodium, phosphorus.

Restricted Cubic Spline analysis (RCS) (with three knots) was used to evaluate the nonlinear associations between CRP and the risk of low muscle mass, the median value of CRP was used as a reference. Two-piecewise Logistic regression analysis model was used to examine the relationship between CRP and low muscle mass and the inflection point. Finally, the authors conducted subgroup analysis to categorize participants into different levels, including age (20 to 60 years/>60 years), gender (male/female), race (Mexican American, Other Races, Non-Hispanic White, Non-Hispanic Black), marital status (married/widowed/divorced/separated, never married, living with a partner), education level (<11th grade, high school, college, above college), vigorous activity (yes/no/unable to do an activity), moderate activity, muscle strengthening activities, waist circumference (cm, 58.5 to 86.5, 86.6 to 96.4, 96.5 to 106.4, 106.5 to 173.4), smoking (yes/no), coronary heart disease (yes/no), congestive heart failure (yes/no), diabetes (yes/no), hypertension (yes/no), stroke (yes/no). Additionally, the authors included interaction analysis to test for heterogeneity among the subgroups. All statistical analyses were performed in R software, version 4.3.1 and *p* < 0.05 was regarded as significant.

## Results

### *Characteristics of the study population*

A total of 13,518 adult participants from NHANES (1999‒2006) were included in this study. Among them, 1592 (11.8 %) had low muscle mass. Participants were grouped according to the muscle mass. Significant differences were observed between the Normal Muscle Mass (NMS) and Low Muscle muscle mass (LMS) groups in terms of age, gender, race, Education level, Marital Status, Smoking, and comorbidity factors such as Hypertension, Diabetes, Arthritis, Congestive heart failure, Coronary heart disease, Stroke, Emphysema, Thyroid disease, Chronic bronchitis, Cancer, Weak/failing kidneys (Table 1); The number of participants in the LMS group who participated in Vigorous activity, Moderate activity, and Muscle strengthening activities was significantly lower compared to the NMS group (Table 1). In addition, the LMS group had higher BMI, systolic blood pressure, and waist circumference compared to the NMS group, but a lower Family poverty income ratio. With the exception of smoking, alcohol use, asthma, liver disease, and diastolic pressure, all other variables showed significant statistical differences between the two groups (Table 1).

**Table 1:** Baseline characteristics of the study population based on muscle mass.

Variables	Overall	NMS	LMS	p
	*n* = 13,518	*n* = 11,926	*n* = 1592	
Age (yr)	49.97 (18.36)	48.23 (17.90)	63.01 (16.38)	<0.001
Gender (%)				0.031
Male	6807 (50.36)	5950 (49.89)	857 (53.83)	
Female	6711 (49.64)	5976 (50.11)	735 (46.17)	
Race (%)				<0.001
Mexican American	3057 (22.61)	2361 (19.80)	696 (43.72)	
Other Race	1059 (7.83)	920 (7.71)	139 (8.73)	
Non-Hispanic White	6774 (50.11)	6076 (50.95)	698 (43.84)	
Non-Hispanic Black	2628 (19.44)	2569 (21.54)	59 (3.71)	
Education level (%)				<0.001
<11th Grade	4285 (31.70)	3433 (28.79)	852 (53.52)	
High School or Equivalent	3213 (23.77)	2898 (24.30)	315 (19.79)	
College or AA degree	3520 (26.04)	3246 (27.22)	274 (17.21)	
College or above	2500 (18.49)	2349 (19.70)	151 (9.48)	
Marital Status (%)				<0.001
Married	7591 (56.15)	6610 (55.43)	981 (61.62)	
Widowed/Divorced/Separated	2966 (21.94)	2514 (21.08)	452 (28.39)	
Never married	2179 (16.12)	2065 (17.32)	114 (7.16)	
Living with partner	782 (5.78)	737 (6.18)	45 (2.83)	
Smoking (%)	7081 (52.38)	6239 (52.31)	842 (52.89)	0.751
Alcohol use (%)	2474 (18.30)	2189 (18.35)	285 (17.90)	0.062
Hypertension (%)	4293 (31.76)	3530 (29.60)	763 (47.93)	<0.001
Diabetes (%)	1337 (9.89)	1019 (8.54)	318 (19.97)	<0.001
Asthma (%)	1516 (11.21)	1335 (11.19)	181 (11.37)	0.868
Arthritis (%)	3476 (25.71)	2835 (23.77)	641 (40.26)	<0.001
Congestive heart failure (%)	414 (3.06)	305 (2.56)	109 (6.85)	<0.001
Coronary heart disease (%)	1075 (7.95)	811 (6.80)	264 (16.58)	<0.001
Stroke (%)	435 (3.22)	320 (2.68)	115 (7.22)	<0.001
Emphysema (%)	256 (1.89)	192 (1.61)	64 (4.02)	<0.001
Thyroid disease (%)	579 (4.28)	499 (4.18)	80 (5.03)	0.010
Chronic bronchitis (%)	854 (6.32)	719 (6.03)	135 (8.48)	<0.001
Liver disease (%)	452 (3.34)	394 (3.30)	58 (3.64)	0.868
Cancer (%)	1120 (8.29)	951 (7.97)	169 (10.62)	<0.001
Weak/failing kidneys (%)	248 (1.83)	188 (1.58)	60 (3.77)	<0.001
Vigorous activity (%)				<0.001
Yes	3926 (29.04)	3742 (31.38)	184 (11.56)	
No	9013 (66.67)	7751 (64.99)	1262 (79.27)	
Unable	579 (4.28)	433 (3.63)	146 (9.17)	
Moderate activity (%)				<0.001
Yes	6197 (45.84)	5702 (47.81)	495 (31.09)	
No	6919 (51.18)	5927 (49.70)	992 (62.31)	
Unable	402 (2.97)	297 (2.49)	105 (6.60)	
Muscle strengthening activities (%)				<0.001
Yes	3322 (24.57)	3140 (26.33)	182 (11.43)	
No	9799 (72.49)	8486 (71.16)	1313 (82.47)	
Unable	397 (2.94)	300 (2.52)	97 (6.09)	
Family poverty income ratio (%)	2.64 (1.61)	2.72 (1.62)	2.10 (1.43)	<0.001
Body mass index, kg/m^2^	28.36 (6.23)	27.97 (6.11)	31.30 (6.35)	<0.001
ASM/BMI	0.80 (0.21)	0.82 (0.20)	0.61 (0.14)	<0.001
Systolic pressure, mmHg	125.11 (32.87)	123.99 (31.96)	133.49 (37.97)	<0.001
Diastolic pressure, mmHg	71.34 (19.55)	71.58 (19.30)	69.52 (21.25)	0.060
Waist Circumference (cm)	97.20 (15.16)	96.21 (14.97)	104.58 (14.56)	<0.001

NMS, Normal Muscle Mass; LMS, Low Muscle Mass; ASM, Appendicular Skeletal Muscle Mass; BMI, Body Mass Index.

Mean ± SD for continuous variables: p-value was calculated by weighted *t*-test.

% For categorical variables: p-value was calculated by weighted Chi-Square test.

Median (interquartile range) for continuous variables: p-value was calculated by Wilcoxon rank-sum test.

### *Laboratory results of the study population*

Table 2 presents the laboratory test results for the two groups. The level of serum CRP in the LMS group was significantly higher than that in the NMS group (0.37 [0.18, 0.74] vs. 0.22 [0.09, 0.50], *p* < 0.001). The LMS group had higher levels of White blood cell count, Neutrophils, Red cell distribution width, aspartate aminotransferase, triglycerides, glucose, serum cholesterol, blood urea nitrogen, globulin, and potassium compared to the NMS group (all *p* < 0.05). There were no statistically significant differences between the two groups in terms of Lymphocyte number, Red cell count, Hemoglobin, Platelet count, Creatinine, and Sodium (all *p* > 0.05).

**Table 2:** Laboratory test results.

Variables	Overall	NMS	LMS	p
	*n* = 13,518	*n* = 11,926	*n* = 1592	
C-reactive protein(mg/dL)	0.22 [0.09, 0.50]	0.20 [0.08, 0.46]	0.37 [0.18, 0.74]	<0.001
White blood cell count (1000 cells/µL)	7.18 (2.31)	7.13 (2.33)	7.55 (2.14)	<0.001
Lymphocyte number (1000 cells/µL)	2.13 (1.24)	2.14 (1.29)	2.10 (0.80)	0.212
Neutrophils (1000 cells/µL)	4.25 (1.63)	4.20 (1.62)	4.61 (1.69)	<0.001
Red cell count (million cells/µL)	4.73 (0.51)	4.73 (0.51)	4.70 (0.50)	0.283
Hemoglobin (g/dL)	14.39 (1.50)	14.39 (1.50)	14.39 (1.48)	0.119
Platelet count (1000 cells/µL)	267.35 (69.08)	267.60 (68.33)	265.51 (74.45)	0.493
Red cell distribution width (%)	12.78 (1.18)	12.75 (1.16)	13.02 (1.34)	<0.001
Albumin (g/L)	43.01(3.35)	43.14 (3.34)	42.06 (3.30)	<0.001
Alanine Aminotransferase (U/L)	21^,^^16^,^29^	21^,^^16^,^29^	21^,^^17^,^30^	0.016
Aspartate Aminotransferase (U/L)	23^,^^19^,^27^	22^,^^19^,^27^	23^,^^20^,^28^	0.038
Creatinine (µmoL/L)	70.7 [61.9, 88.4]	70.7 [61.9, 88.4]	70.7 [61.9, 88.4]	0.497
Triglycerides (mmoL/L)	1.30 [0.87, 1.95]	1.25 [0.85, 1.90]	1.58 [1.16, 2.31]	<0.001
Glucose (mmoL/L)	5.48 (1.95)	5.39 (1.83)	6.13 (2.60)	<0.001
Serum Cholesterol (mmoL/L)	5.19 (1.09)	5.17 (1.08)	5.33 (1.13)	<0.001
Blood Urea Nitrogen (mmoL/L)	4.96 (2.12)	4.87 (2.00)	5.65 (2.76)	<0.001
Total Calcium (mmoL/L)	2.37 (0.10)	2.37 (0.10)	2.36 (0.10)	0.007
Globulin (g/L)	30.70 (4.62)	30.61 (4.62)	31.33 (4.57)	<0.001
Potassium (mmoL/L)	4.05 (0.35)	4.05 (0.35)	4.09 (0.37)	0.010
Sodium (mmoL/L)	139.11 (2.46)	139.10 (2.45)	139.22 (2.56)	0.309
Phosphorus (mmoL/L)	1.18 (0.18)	1.18 (0.18)	1.17 (0.18)	0.036

NMS, Normal Muscle Mass; LMS, Low Muscle Mass.

Mean ± SD for continuous variables: p-value was calculated by weighted *t*-test.

% for categorical variables: p-value was calculated by weighted Chi-Square test.

Median [interquartile range] for continuous variables: p-value was calculated by Wilcoxon rank-sum test.

### *Association between CRP and low muscle mass*

Through weighted logistic regression analysis, a significant association was observed between elevated CRP levels and an increased risk of low muscle mass (Table 3). When CRP was treated as a continuous variable and fully adjusted for covariates (Model 3), a positive association persisted between CRP and the risk of low muscle mass. Specifically, for every unit increase in serum CRP level, the risk of low muscle mass increased by 7 % (OR = 1.07, 95 % CI 1.01‒1.17, *p* = 0.016) (Table 3).

**Table 3:** Weighted logistic regression analysis of CRP and low muscle mass.

	Model 1		Model 2		Model 3	
	OR (95 % CI)	p	OR (95 % CI)	P	OR (95 % CI)	p
**Continuous**	1.21 (1.16, 1.27)	<0.001	1.18 (1.12, 1.24)	<0.001	1.07 (1.01, 1.14)	0.016
**Categories**						
Tertile 1	Ref.		Ref.		Ref.	
Tertile 2	2.21 (1.82, 2.67)	<0.001	1.75 (1.44, 2.13)	<0.001	1.39 (1.13, 1.72)	0.002
Tertile 3	3.27 (2.72, 3.91)	<0.001	2.55 (2.11, 3.07)	<0.001	1.84 (1.50, 2.27)	<0.001
Tertile 4	4.55 (3.82, 5.43)	<0.001	3.85 (3.21, 4.63)	<0.001	2.43 (1.95, 3.02)	<0.001
p for trend		<0.001		<0.001		<0.001

Tertile 1: 0.01‒0.09 mg/dL; Tertile 2: 0.10‒0.22 mg/dL; Tertile 3: 0.23‒0.50 mg/dL; Tertile 4: 0.51‒29.6 mg/dL.

In sensitivity analysis, utilizing CRP as a categorical variable, a dose-response relationship was observed. Participants in higher quartiles of CRP exhibited a progressively elevated risk of low muscle mass compared to those in the lowest quartile (p for trend < 0.001). Notably, individuals in the highest CRP quartile demonstrated a statistically significant 143 % increase in the risk of low muscle mass (OR = 2.43; 95 % CI 1.95‒3.02; *p* < 0.001) (Table 3).

### *Restricted cubic spline and threshold effect analyses*

**Fig. 2:**
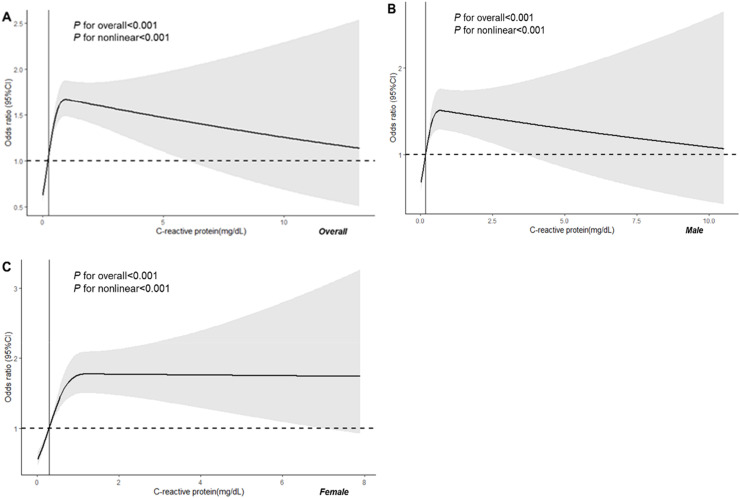
Restricted cubic spline analysis of the relationship between C-reactive protein and low muscle mass. Overall (A), Male (B), Female (C).

To further investigate the relationship between CRP and low muscle mass, the authors used smooth curve fitting and found an inverse J-shaped association after fully adjusting for covariates (Fig. 2); after using two-piecewise linear regression, the results showed that the inflection point was 0.273 mg/dL. When serum CRP level was below 0.273 mg/dL, the risk of low muscle mass decreased with increasing CRP (OR = 0.74; 95 % CI 0.70‒0.77; *p* < 0.001). However, when serum CRP level exceeded 0.273 mg/dL, the risk of low muscle mass increased with the elevation of CRP (OR = 1.36; 95 % CI 1.29–1.43; *p* < 0.001) (Table 4).

**Table 4:** Analysis of the threshold effect of CRP on low muscle mass by two-piece linear regression model.

Inflection point	OR (95 % CI)	p
**Overall**		
≤ 0.273 mg/dL	0.74 (0.70, 0.77)	<0.001
> 0.273 mg/dL	1.36 (1.29, 1.43)	<0.001
Log likelihood ratio tests		<0.001
**Male**		
≤ 0.172 mg/dL	0.69 (0.64, 0.74)	<0.001
> 0.172 mg/dL	1.46 (1.35, 1.57)	<0.001
Log likelihood ratio tests		<0.001
**Female**		
≤ 0.296 mg/dL	0.77 (0.73, 0.83)	<0.001
> 0.296 mg/dL	1.30 (0.73, 0.83)	<0.001
Log likelihood ratio tests		<0.001

Gender-specific analysis revealed that in males when the serum CRP level was ≤ 0.172 mg/dL, there was a significant decrease in the odds of low muscle mass (OR = 0.69, 95 % CI 0.64‒0.74, *p* < 0.001). Conversely, when the serum CRP level exceeded 0.172 mg/dL, there was a significant increase in the odds of low muscle mass (OR = 1.46, 95 % CI 1.35‒1.57, *p* < 0.001). In females, when the serum CRP level was ≤ 0.296 mg/dL, there was a significant decrease in the odds of low muscle mass (OR = 0.77, 95 % CI 0.73‒0.83, *p* < 0.001). However, when the serum CRP level exceeded 0.296 mg/dL, there was no significant difference in the odds of low muscle mass (OR = 1.30, 95 % CI 0.73‒0.83, *p* < 0.001). These results indicate a clear association between serum CRP levels and the risk of low muscle mass (Table 4).

### *Subgroup analyses*

**Fig. 3:**
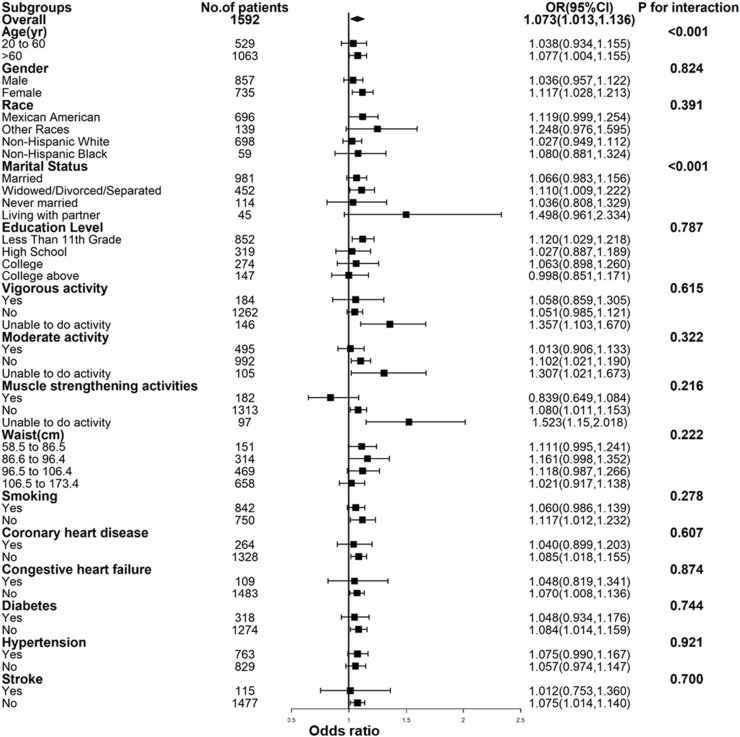
Subgroup analysis for the association between C-reactive protein and low muscle mass by weighted logistic regression analysis.

To evaluate the stability of the association between CRP and low muscle mass across different subgroups of participants, the authors conducted a subgroup analysis. Interaction tests revealed no statistically significant differences in the correlation between CRP and the risk of low muscle mass among different subgroups (Fig. 3). This indicates that factors such as gender (male/female), race (Mexican American/Other Hispanic/Non-Hispanic White/Non-Hispanic Black/Other), educational level (<11th Grade, High School or Equivalent, College or AA degree, College or above), smoking status (yes/no), congestive heart failure (yes/no), stroke (yes/no), renal weakness/failure (yes/no), coronary heart disease (yes/no), diabetes (yes/no), hypertension (yes/no), vigorous activity(yes/no/unable), moderate activity (yes/no/unable), and muscle strengthening activities (yes/no/unable) did not have a significant impact on this positive correlation (all p for interaction values > 0.05). However, there were significant interactions observed in terms of age (20‒60 years/ > 60 years) and marital status (married, widowed/divorced/separated, never married, living with partner) (all p for interaction values < 0.001).

## Discussion

In this comprehensive survey involving 13,518 adult participants from the United States, the authors found a significant correlation between serum CRP levels and the prevalence of low muscle mass. Through detailed subgroup analysis and interaction testing, the authors explored these findings and revealed similar trends in this association. A smooth curve fitting analysis demonstrated an inverse J-shaped relationship, with a two-piecewise linear regression indicating an inflection point at 0.273 mg/dL. When serum CRP level was below 0.273 mg/dL, the risk of low muscle mass decreased with increasing CRP. However, when serum CRP level exceeded 0.273 mg/dL, the risk of low muscle mass increased with the elevation of CRP. These findings validate and deepen the initial hypothesis proposed in this study, highlighting the complex interaction between CRP and low muscle mass in adults. This provides crucial insights for advancing our understanding of the inflammatory processes involved in low muscle mass.

Based on existing literature, the present study represents the largest sample size investigation to date exploring the relationship between C-Reactive Protein (CRP) and low muscle mass in adults. Previous studies, such as the work conducted by Shokri-Mashhadi and others, have revealed an independent association between muscle strength impairment and circulating C-Reactive Protein (CRP) and high-sensitivity C-Reactive Protein (hs-CRP), noting a significant negative correlation between CRP and hs-CRP levels and muscle strength.^14^ However, despite providing valuable insights, this study did not provide specific predictive values for the relationship between CRP and low muscle mass, limiting the understanding of this relationship to some extent. On the other hand, a study by Lee WJ et al.^15^ involving 1582 sarcopenic patients provided us with additional clues. They found that high levels of hs-CRP were independently associated with low muscle mass, with a more significant trend observed among female participants. This discovery adds to the evidence linking inflammatory markers such as CRP to the development of low muscle mass, further supporting the role of inflammation in the pathogenesis of this condition. Moreover, Lobo has noticed a negative correlation between CRP levels and muscle strength in elderly male individuals, while no such correlation was observed in female participants.^22^ This finding suggests that the relationship between inflammation and muscle strength in the elderly may be gender-specific, emphasizing the importance of considering gender differences when studying the impact of inflammation on muscle function in older adults. In this study, weighted logistic regression analysis was conducted with gender as a subgroup, and the results revealed a positive correlation between CRP and the risk of low muscle mass. Notably, the Odds Ratio value in female participants was significantly higher than in males. Although no inter-group interaction was observed, no statistically significant difference was found in males. This finding is consistent with the conclusion of Lobo's research.

The research has found that low muscle mass also has a certain prevalence among middle-aged and young adults. Silva's study observed that even among young individuals, changes in all parameters had a negative impact on strength, mass, and muscle function.^23^ Muscle mass can be considered an important risk factor for functional disability and should be taken into account when assessing low muscle mass. This underscores the importance of early recognition and intervention to prevent and manage low muscle mass, even in younger age groups. Therefore, in this study, adults over 20 years old were selected as the research subjects to comprehensively analyze the association between CRP and the risk of low muscle mass, and a weighted analysis was performed. The results were true, reliable, and representative.

The most intriguing finding of the present study, contrasting with previous research, is the discovery of a nonlinear association between serum CRP and the risk of low muscle mass in adults, specifically exhibiting an inverse J-shaped pattern. The inflection point was identified at 0.273 mg/dL. Below this threshold, as CRP levels increase, the risk of low muscle mass decreases. However, once CRP values surpass this threshold, the risk of low muscle mass rises significantly. This novel observation provides a more nuanced understanding of the relationship between CRP and low muscle mass, with potential implications for future research and clinical management of this condition.

When serum CRP levels are below 0.273 mg/dL, the potential mechanism underlying the negative correlation between CRP and the risk of low muscle mass in adults has been analyzed by integrating reports from the literature. Firstly, Inflammation and Muscle Health: While inflammation is generally associated with negative effects on muscle health, including low muscle mass, it's important to consider that inflammation also plays a role in muscle repair and regeneration.^24^ Low levels of CRP may indicate minimal systemic inflammation, which could potentially be beneficial for maintaining muscle health and reducing the risk of low muscle mass.^25^ Secondly, Insulin Sensitivity: Low CRP levels may also be associated with better insulin sensitivity.^26^ Insulin resistance has been linked to muscle wasting and low muscle mass, so improved insulin sensitivity could help mitigate these effects and contribute to better muscle health.^27^ Thirdly, Healthy Lifestyle Factors: Low CRP levels may also be indicative of a generally healthier lifestyle, including regular exercise and a balanced diet, both of which are important for maintaining muscle mass and function.^28^ These lifestyle factors can have protective effects against low muscle mass. Fourthly, Muscle Preservation: Low CRP levels may be associated with decreased muscle protein breakdown and improved muscle preservation.^29^ This could be due to various factors, including reduced systemic inflammation and better regulation of muscle metabolism. Fifthly, Genetic Factors: Genetic variations may also contribute to the observed correlation between CRP levels and low muscle mass.^30^ Certain genetic factors can influence both inflammation levels and muscle health, impacting the relationship between CRP and low muscle mass. Additionally, considering the complexity of low muscle mass and inflammation, it's crucial to interpret findings within the context of broader physiological and clinical factors.

However, when the level of CRP exceeds 0.273 mg/dL, it is positively correlated with the risk of adult low muscle mass. This association can be attributed to multiple mechanisms: chronic inflammation leads to increased muscle protein breakdown and impaired regeneratio.^31^ High CRP levels directly promote muscle breakdown metabolism and further exacerbate muscle loss by upregulating pro-inflammatory cytokines.^11^ A higher inflammatory state can cause insulin resistance, hormonal imbalance, oxidative stress, and endothelial dysfunction. Insulin resistance impairs muscle protein synthesis and increases the risk of low muscle mass.^32^ Hormonal imbalance accelerates muscle loss.^33^ Oxidative stress impairs muscle cell function.^34^ Impaired muscle regeneration leads to increased muscle loss; Endothelial dysfunction reduces muscle blood flow, hindering muscle repair and growth.^35^ These mechanisms collectively indicate the multifactorial nature of low muscle mass, emphasizing the crucial role of inflammation and its related pathways in prevention and management.

This study possesses profound practical significance. Firstly, it reveals the negative impact of inflammation on muscle quality in American adults, reflected by the level of serum CRP, emphasizing the social importance of inflammation as a potential risk factor for muscle dysfunction, which concerns overall health and quality of life. Secondly, healthcare professionals, including doctors, nutritionists, and physical therapists, can benefit from this research by monitoring CRP levels to assess muscle health, thus enabling early identification and intervention for muscle-related disease risks. Finally, this study provides prevention and treatment strategies for inflammation-related muscle dysfunction in the healthcare system. By identifying critical CRP turning points, the healthcare system can develop effective screening and treatment plans to mitigate the damage of inflammation to muscle health, thereby reducing related health issues and medical costs.

The present study has several limitations that are worth noting. Firstly, smoking, alcohol use, physical activity, and disease status were self-reported, which may be subject to misclassification and recall bias. This could potentially lead to an overestimation or underestimation of the association between CRP and low muscle mass. Additionally, this study is cross-sectional, meaning that the relationship between CRP and low muscle mass cannot be interpreted as a direct causal link. Finally, the participants in the present study were adults from the United States, and given the unique manifestations of race and genetics, generalizations to other regions should be made with caution. To address these limitations, future studies could consider using more objective measures for smoking, physical activity, and disease status, such as biomarker testing or medical records, to reduce the potential for misclassification and recall bias. Longitudinal studies would also be beneficial in establishing a more robust understanding of the causal relationship between CRP and low muscle mass. Finally, when extrapolating the findings of this study to other populations, it is important to consider the potential influence of racial and genetic differences on the association between CRP and low muscle mass.

## Conclusion

In summary, the present study involving 13,518 adult participants from the United States revealed a significant correlation between serum CRP levels and low muscle mass prevalence. The authors found an inverse J-shaped association, with an inflection point at 0.273 mg/dL. Below this threshold, increasing CRP levels were associated with decreased low muscle mass risk, while above it, the risk increased significantly. These findings deepen our understanding of the complex interplay between CRP and low muscle mass in adults. Future research should focus on longitudinal studies with objective measures to further elucidate the causal relationship between CRP and low muscle mass and consider potential racial and genetic differences in different populations.

## Consent for publication

Not applicable.

## Authors’ contributions

Conceptualization: Ruzheng Lin and Ying Chen.

Formal analysis: Kai Liu.

Funding acquisition: Ying Chen.

Supervision: Kai Liu.

Writing-original draft: Ruzheng Lin, Ying Chen, Kai Liu.

Writing-review & editing: Ying Chen and Kai Liu.

## Funding

Project supported by Hainan Province Clinical Medical Center (2021276).

## Declaration of competing interest

The authors declare no conflicts of interest.
